# Overexpression of Human SOD1 Leads to Discrete Defects in the Cerebellar Architecture in the Mouse

**DOI:** 10.3389/fnana.2017.00022

**Published:** 2017-03-29

**Authors:** Pegah Afshar, Niloufar Ashtari, Xiaodan Jiao, Maryam Rahimi-Balaei, Xiaosha Zhang, Behzad Yaganeh, Marc R. Del Bigio, Jiming Kong, Hassan Marzban

**Affiliations:** ^1^Department of Human Anatomy and Cell Science, The Children's Hospital Foundation University of Manitoba, Rady Faculty of Health Sciences, Max Rady College of Medicine, University of ManitobaWinnipeg, MB, Canada; ^2^Program in Physiology and Experimental Medicine, Hospital for Sick Children and University of TorontoToronto, ON, Canada; ^3^Department of Pathology, Faculty of Medicine, University of ManitobaWinnipeg, MB, Canada

**Keywords:** cerebellum, calbindin, Purkinje cell, stripes, cerebellar vermis, transgenic mice

## Abstract

The human superoxide dismutase 1 (*SOD1*) gene is responsible for neutralizing supercharged oxygen radicals within the cell. Mutation in SOD1 gene causes amyotrophic lateral sclerosis (ALS). Recent studies have shown involvement of the cerebellum in ALS, although the cerebellar contribution in *SOD1* transgenic mice remains unclear. Using immunohistopathology, we investigated the Purkinje cell phenotype in the vermis of the *SOD1* transgenic mice cerebellum. Calbindin 1 (Calb1) and three well-known zone and stripe markers, zebrin II, HSP25, and PLCβ4 have been used to explore possible alteration in zone and stripe. Here we show that Calb1 expression is significantly reduced in a subset of the Purkinje cells that is almost aligned with the cerebellar zones and stripes pattern. The Purkinje cells of *SOD1* transgenic mice display a pattern of Calb1 down-regulation, which seems to proceed to Purkinje cell degeneration as the mice age. The onset of Calb1 down-regulation in Purkinje cells begins from the central zone and continues into the nodular zone, however it has not been observed in the anterior and posterior zones. In a subgroup of *SOD1* transgenic mice in which gait unsteadiness was apparent, down-regulation of Calb1 is seen in a subset of PLCβ4^+^ Purkinje cells in the anterior zone. These observations suggest that the Calb1^−^ subset of Purkinje cells in the anterior zone, which receives somatosensory input, causes unsteady gait. Our data suggest that human SOD1 overexpression leads to Calb1 down-regulation in the zone and strip pattern and raise the question of whether SOD1 overexpression leads to Purkinje cells degeneration.

## Introduction

The human superoxide dismutase 1 (*SOD1*) gene is located on chromosome 21 and functions to produce the enzyme Cu/Zn SOD1, which neutralizes superoxide (oxygen) radicals within cells. Mutations in this gene may cause the enzyme to gain toxic properties that are associated with rare familial motor neuron disease/amyotrophic lateral sclerosis (ALS) in humans (Rotunno and Bosco, 2013). Patients with ALS experience a progressive loss of motor neurons in the spinal cord and brain stem, and may become completely paralyzed toward the later stages of the disease (Gordon, 2013). ALS was traditionally considered to be a pure motor disorder (van der Graaff et al., 2009), but is now considered to be a multisystem neurodegenerative disease including frontal lobe, basal ganglia, and substantia nigra, and the cerebellum (Cellura, 2011; Mochizuki et al., 2012; Williams, 2013). The involvement of the cerebellum in ALS was recently reviewed by Prell and Grosskreutz (2013). Mouse models of human *SOD1* mutations are valuable for understanding multisystem involvement and they provide significant insights into the mechanisms of ALS (Pioro and Mitsumoto, 1995). Evidence suggests that the sensory and spino-cerebellar pathways are involved, as well as neuronal groups within the substantia nigra and the hippocampal dentate granule layers (Cotterill, 2001; Prell and Grosskreutz, 2013). In *SOD1-G93A* Tg mice model for ALS (*SOD1*^*G93A*^). The most prominent alterations of tau expression were reported in the cerebellum (Baranczyk-Kuzma et al., 2007).

The cerebellum is implicated in receiving sensory input and integrating it into motor output and non-motor functions (Timmann et al., 2010; Popa et al., 2014). The three-layered cerebellar cortex is comprised of Purkinje cells, granule cells and GABAergic interneurons (Voogd and Glickstein, 1998). Purkinje cells are the sole output neurons of the cerebellar cortex and they are primarily responsible for the complex topography that results in a set of zones and stripes in the cerebellum (Voogd and Glickstein, 1998; Apps and Hawkes, 2009; Vibulyaseck et al., 2015). The mouse cerebellum is divided into four transverse zones: the anterior zone (AZ; lobules I–V), the central zone (CZ; lobules VI–VII; Marzban et al., 2008; Sawada et al., 2008), the posterior zone (PZ; lobules VIII–dorsal IX) and the nodular zone (NZ; ventral lobule IX and lobule X; Brochu et al., 1990; Eisenman and Hawkes, 1993; Ozol et al., 1999; Sillitoe and Hawkes, 2002; Marzban and Hawkes, 2011; Bailey et al., 2013, 2014). Each zone is further subdivided into parasagittal stripes. The most intensively studied markers of parasagittal stripes are zebrin II (ZII) and phospholipase Cβ4 (PLCβ4; e.g., Marzban et al., 2003, 2007; Kim et al., 2009). The AZ is subdivided into ZII^+^ stripes, separated by ZII^−^ stripes, which are PLCβ4^+^ (Sarna et al., 2006; Marzban et al., 2007). The same pattern is observed in the PZ, although the CZ and the NZ have uniform ZII expression or are negative for PLCβ4 (Sillitoe and Hawkes, 2002; Marzban and Hawkes, 2011). The Purkinje cells in the CZ and NZ are further subdivided into parasagittal stripes by expression of the small heat shock protein 25 (HSP25). The CZ shows five parasagittal stripes with HSP25 in the vermis, one midline and two on each side. In the NZ, five parasagittal bands of HSP25-immunoreactive Purkinje cells appear symmetrically about the midline (Armstrong et al., 2000; Bailey et al., 2014). The complexity of the compartmentation in the cerebellum does not end with ZII, PLCβ4, and HSP25 markers; other markers differentiate several narrow stripes within the ZII^+^ and PLCβ4^+^ regions (Akintunde and Eisenman, 1994; Armstrong et al., 2000; Marzban et al., 2007; Bailey et al., 2014). The selective gene expression in different regions and the patterning of Purkinje cells are indicators of compartmentation and different insult vulnerability levels. Therefore, Purkinje cell degeneration, in most cases, appears in a strip-like pattern that corresponds to a specific region within the cerebellum (Sarna and Hawkes, 2003). For example, in murine models of Niemann-Pick disease type C (NPC), Purkinje cell loss first occurs with ZII immunonegative stripes in the AZ, and then progresses to the ZII immunopositive Purkinje cells. However, the Purkinje cells that express HSP25 are more resistant to degeneration than those lacking this protein (Sarna et al., 2003; Duffin et al., 2010).

Wild type human *SOD1* transgenic mice (wt *SOD1* Tg mice) have been used as controls for many experimental studies concerning ALS with the assumption that wt human SOD1 has no deleterious effects to neurons (Furukawa, 2012). However, posttranscriptional modification of wt SOD1 occurs with aging and has been shown to be toxic to neurons (Furukawa, 2012). Here, we hypothesize that the wt SOD1 expression has toxic effect on cerebellar Purkinje cell with pattern parasagittal phenotype. The adult wt *SOD1* Tg mice cerebellum is used to study the Purkinje cell phenotype using calbindin 1 (Calb1), calcium-binding protein encoded by the gene (*Calb1*), along with zone and stripe markers. This study shows that the transgenic *SOD1* gene and/or its gene product interfere with cellular mechanisms in the Purkinje cells. In contrast to the expected observation that Calb1 is expressed uniformly in all Purkinje cells, Calb1 expression is significantly down-regulated in the CZ and NZ of wt *SOD1* Tg mice. Calb1 immunopositive Purkinje cells have the same expression pattern as that of HSP25 in the CZ and NZ. This study will further our understanding of the wt *SOD1* Tg mice as a model of ALS, determine the effect of the *SOD1* gene on Purkinje cells and show an expression pattern of Calb1 down-regulation and may proceed to degeneration in subset of Purkinje cell in wt *SOD1* Tg mice.

## Materials and methods

### Animal maintenance

All animal procedures for this study were performed in accordance with Canadian Council of Animal Care guidelines and approved by the Animal Care Review Committee of the University of Manitoba. WT *SOD1* Tg mice (B6.Cg-Tg (SOD1)2Gur/J, JAX Stock No. 002299) were obtained from Jackson's Laboratory, by JAX's description, this line carries the normal allele of the human *SOD1* gene. Originally published as N1029, it expresses the same SOD1 protein level as the transgenic strain carrying the SOD1^*^G93A transgene (002726), even though the copy number in the SOD1^*^G93A transgenic is higher (Gurney et al., 1994; Dal Canto and Gurney, 1995). In this study, we observed in the offspring from 15 litters, 78 subjects did carry and 73 did not carry the wt human SOD1 Tg (controls). We have used cerebellum of the 7 wt *SOD1* Tg mice at 5 month, and 10 wt *SOD1* Tg mice at 8 month old (included 2 with unsteady gait) with an equal number of controls.

### Perfusion and sectioning

All mice were deeply anesthetized with 20% isoflurane, USP (Baxter Co. Mississauga, Ontario, Canada) in propylene glycol (Sigma-Aldrich Canada Co., Ontario, Canada) using a desiccator. The mice were transcardially perfused with 15 ml of 0.1 M phosphate buffer saline (PBS; pH 7.4) and 30 ml of 4% paraformaldehyde (PFA) in PBS. The brains were removed and post-fixed in 4% PFA at 4°C for at least 24 h. The cerebellum was removed and cryoprotected using 10% (2 h), 20% (2 h), and 30% (24 h) sucrose solution in PBS. The cerebella were then frozen in clear frozen section compound (VWR, Mississauga, Ontario, Canada) at −80°C for 30 min. Transverse sections of the cerebellum were serially cut at a 30 μm thickness using a −20°C cryostat and collected in PBS for free-floating immunohistochemistry.

### Human brain sections

Human brain samples were obtained from three ALS patients not known to have *SOD1* mutation and from 3 age- and sex-matched control cases with no evidence of neurological disease. ALS cases were family permission autopsies including consent for research. Controls were acquired under University of Manitoba Health Research Ethics Board protocol H2013:217. The autopsies on all cases were conducted 24–48 h after death using standard safety precautions. The bodies were refrigerated at 4°C in the interim. Samples were fixed in 10% formalin for 10–14 days; tissue samples were dehydrated in graded alcohols and embedded in paraffin. In conjunction with appropriate clinical histories, ALS diagnosis was made by histologic examination of the spinal cord including demonstration of skein-like ubiquitin immunoreactive inclusions in residual motor neurons. For this study, sections of posterior cerebellum were cut at 5 μm thickness in the transverse plane and mounted on glass slides.

### Single staining immunohistochemistry

Immunohistochemistry was performed on cerebellar sections, as previously described (Chung et al., 2009; Marzban et al., 2012). Cerebellar sections were washed with 0.1 M PBS three times for 5 min. The sections were then incubated in 0.3% peroxidase for 20 min, washed with PBS three times for 5 min, blocked with blocking buffer containing 10% normal goat serum in 0.1 M PBS and 0.05% Triton-X100 (Fisher Scientific) for 1 h and incubated in primary antibody in the blocking solution at room temperature. The following primary antibodies were used: rabbit polyclonal anti-Calb1 (calbindin D-28K, anti-CaBP; diluted 1:1000, Swant Inc., Bellinzona, Switzerland), mouse monoclonal anti-Calb1 (diluted 1:1000, Swant Inc., Bellinzona, Switzerland), anti-zebrin II (ZII; diluted 1:200, a gift from Dr. Richard Hawkes, University of Calgary, Calgary, Alberta, Canada), anti-phospholipase Cβ4 (PLCβ4; diluted 1:100, Abcam Inc.: ab103279), anti-SOD1, Rabbit polyclonal [diluted 1:500, Santa Cruz Biotechnology Inc., Dallas, Texas, USA, anti-SOD1 (FL-154) SC-11407] and goat polyclonal anti-SOD1 [diluted 1:500, Santa Cruz Biotechnology Inc., Santa Cruz, USA, anti-SOD1(N-19) sc-8636]and anti-rabbit small heat shock protein 25 (HSP25; diluted 1:1000; StressGen, Victoria BC, Canada). The sample was washed three times with 0.1 M PBS for 5 min, incubated with horseradish peroxidase (HRP)-conjugated goat anti-rabbit or HRP-conjugated goat anti-mouse antibody (diluted 1:500, Millipore) in blocking buffer for 1 h at room temperature. They were then washed with PBS, stained with diaminobenzidine (DAB, 0.5 mg/ml), washed with PBS, and mounted on a slide. The sections were then dehydrated in alcohol series and xylene, and mounted with mounting medium Krystalon™ (Millipore EMD) and cover-slipped.

### Double staining immunohistochemistry

Double labeling of cerebellar sections was performed similar to the single staining, as described above. The two primary antibodies were applied to the sample in the same buffer solution. Secondary antibodies included Alexa Fluor 546-conjugated goat anti-rabbit Ig and Alexa Fluor 488-conjugated goat anti-mouse Ig (diluted 1:1000, Molecular Probes Inc., Eugene, OR, USA). Sample was mounted onto a slide after a secondary antibody wash (chromogen or dehydration steps are not applicable). Sample was then mounted using FluorSave Reagent (Calbiochem, La Jolla, CA, USA #345789).

### Nissl staining

Nissl staining was performed as previously described (Ezzi et al., 2007), with some modifications. Free-floating frozen sections that were dried on a slide were rehydrated using 70% ethanol, 50% ethanol and double distilled H_2_O, and stained in 0.1% cresyl violet solution, followed by differentiation in acetic acid ethanol solution and dehydration using an ethanol series. The sample was then cleared and mounted using mounting medium Krystalon™ (Millipore EMD).

### Primary culture of dissociated cerebellum

Primary cerebellum cultures were prepared from embryonic (E) day 18 CD1 mice and cells were maintained for 21 days *in vitro* (DIV = 21; Marzban and Hawkes, 2007; Bailey et al., 2013). Briefly, the entire cerebellum was removed and immediately placed into ice-cold Ca^2+^/Mg^2+^ free Hank's balance salt solution (HBSS) containing gentamicin (10 μg/ml) and glucose (6 mM). The cerebella were incubated at 34°C for 12 min in HBSS containing 0.1% trypsin. After washing, the cerebella were gently triturated in HBSS containing DNase I (5 U/ml) and 12 mM MgSO_4_ until the cell mass was no longer visible. The cells were collected by centrifugation (1,200 rpm, 4°C for 5 min) and re-suspended in seeding medium (1:1 Dulbecco's modified Eagle's medium and F12) supplemented with putrescine (100 μM), sodium selenite (30 nM), L-glutamine (1.4 mM), gentamicin (5 μg/ml) and 10% heat-inactivated fetal bovine serum. The cell suspensions were seeded on poly-L-ornithine coated glass coverslips (12 mm) at a density of 5 × 10^6^ cells/ml, with each coverslip placed into a well of a 24-well plate. After incubation for 6–8 h in a CO_2_ incubator (100% humidity, 37°C, 5% CO_2_), 500 μl of culture medium supplemented with transferrin (200 μg/ml), insulin (20 μg/ml), progesterone (40 nM), and triiodothyronine (0.5 ng/ml) was added to each culture well. Every 7 days, half of the medium in each dish was replaced with fresh medium that was additionally supplemented with cytosine arabinoside (4 μM) and bovine serum albumin (100 μg/ml).

### Imaging and figure preparation

For bright field microscopy, images were captured using Zeiss Axio Imager M2 microscope (Zeiss, Toronto, ON, Canada). Images were than analyzed with a Zeiss Microscope Software (Zen Image Analyses software; Zeiss, Toronto, ON, Canada). For fluorescence microscopy of the entire cerebellum sections, a Zeiss Lumar V12 Fluorescence stereomicroscope (Zeiss, Toronto, ON, Canada) equipped with camera was applied to capture the images. Images were then analyzed using Zen software. For high magnification fluorescence microscopy, a Zeiss Z1 and Z2 Imager and a Zeiss LSM 700 confocal microscope (Zeiss, Toronto, ON, Canada) equipped with camera and Zen software were used to capture and analyze images. Images were cropped, corrected for brightness and contrast, and assembled into montages using Adobe Photoshop CS5 Version 12. ImageJ/Fiji software was used to calculate the integrated densities of immunostaining in the region of interest in each slide (Schindelin et al., 2012; Facchinello et al., 2016). Briefly, threshold used to highlight the area of interest within the entire image; consequently selection tools were used to select the specific smaller area of interest. The integrated density was measured within multiple selected area of image. The value of density were normalized by the area and illustrated as a bar graph using GraphPad Prism 5.0 software.

### Statistical analysis

All values were expressed as mean ± standard error of mean (SEM). Statistical analysis was performed with GraphPad Prism Version 6.0 software (GraphPad Software Inc.). Statistical significance between groups was determined by the unpaired two-tailed Student's *t*-test. *P* < 0.05 were defined as statistically significant.

## Results

### Calbindin 1 (Calb1) was down-regulated in a patterned manner in wt *SOD1* Tg mice

**Figure 1:**
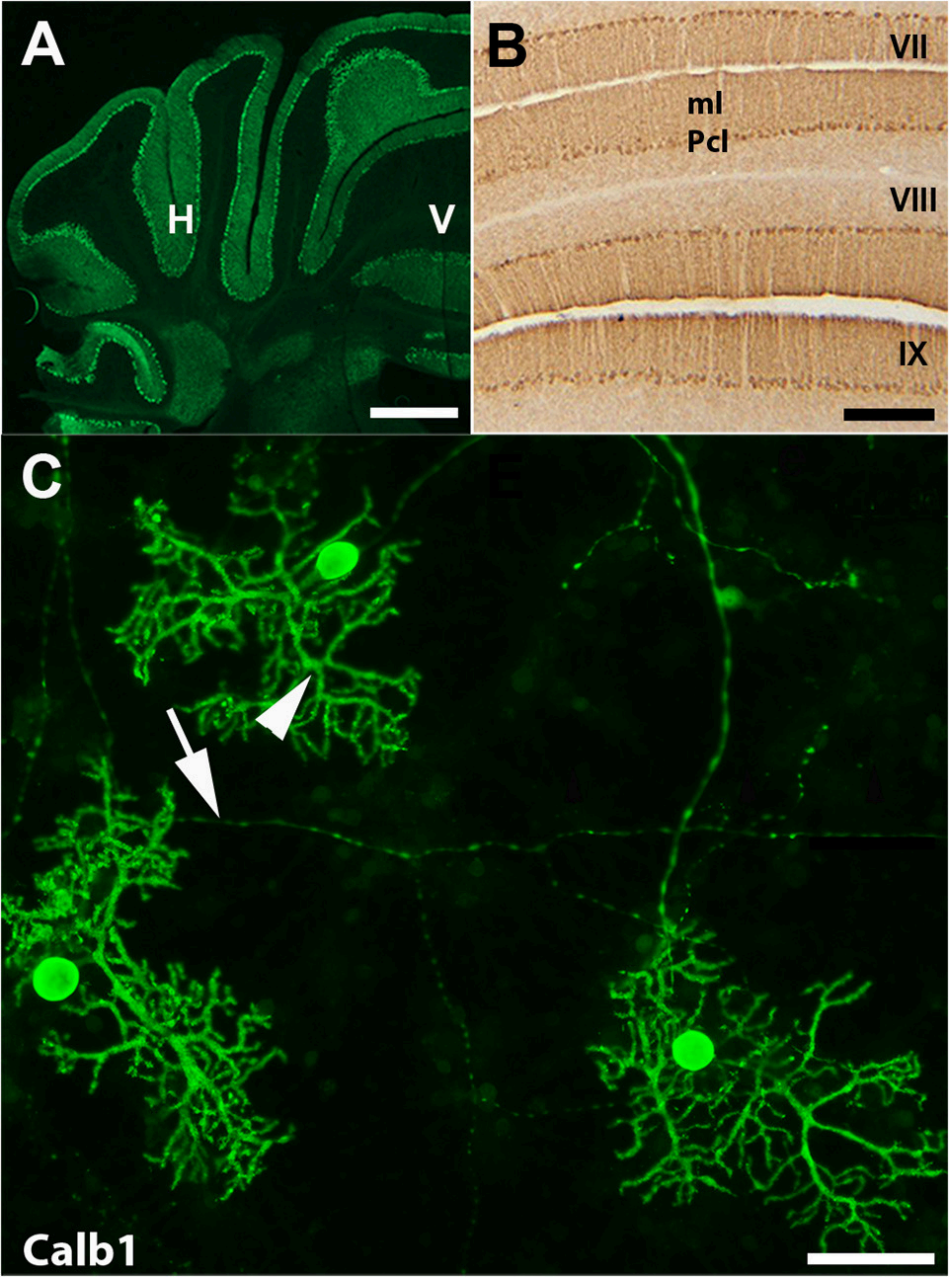
**Calbindin 1 (Calb1) is specific marker for Purkinje cells in the cerebellum. (A,B)** Transverse cryostat sections through the adult control mouse cerebellum immunofluorescent staining using anti-Calb1 show that Purkinje cell soma and dendrites are in the entire cerebellar cortex and show uniform Calb1 expression **(A)**. Panel **(B)** is shown at a higher magnification in lobule VII–IX, which is immunoperoxidase stained with Calb1. **(C)** Purkinje cells from primary dissociated cerebellar culture at embryonic (E) day 18, day *in vitro* (DIV) = 21. Calb1 immunoflourscence staining clearly shows Purkinje cell soma, dendrites (arrow head) and axons (arrow). ml, molecular layer; H, hemisphere; V, vermis. Scale bar = 2 mm in **(A)**; 250 μm in **(B)**; 50 μm in **(C)**.

In the mouse cerebellum, Calb1 was expressed exclusively in Purkinje cells (Figure 1; e.g., Baimbridge et al., 1982; Marzban and Hawkes, 2007). To study expression pattern of Calb1, we applied immunostaining using Calb1 antibody *in vivo* and *in vitro*. Transverse section immunostaining using anti-Calb1 showed that Purkinje cell stomata are arranged in a monolayer pattern in all the cerebellar lobes/lobules with dendrite extensions to the molecular layer and axons to the granular layer (Figures 1A,B). Immunocytochemistry of primary cerebellar cultures prepared from mouse embryos (embryonic day 18; DIV = 21) also showed Calb1 expression in Purkinje cell soma, dendrites, and axons (Figure 1C).

To determine whether human SOD1 is present in Purkinje cells, the immunohistochemistry was performed using anti human SOD1 antibody on 8 month wt *SOD1* Tg mouse cerebella and the human ALS cerebellar samples (Supplementary Figure 1). There was no immunoperoxidase deposit in Purkinje cells of the control mouse cerebellum, while in wt *SOD1* Tg mice cerebellar cortex clear immunoreactivity was observed in the Purkinje cell layer of the entire cerebellum. In human cerebellum, SOD1 immunoreactivity was clearly present in Purkinje cell soma and dendrites of control and ALS samples (Supplementary Figure 1).

**Figure 2:**
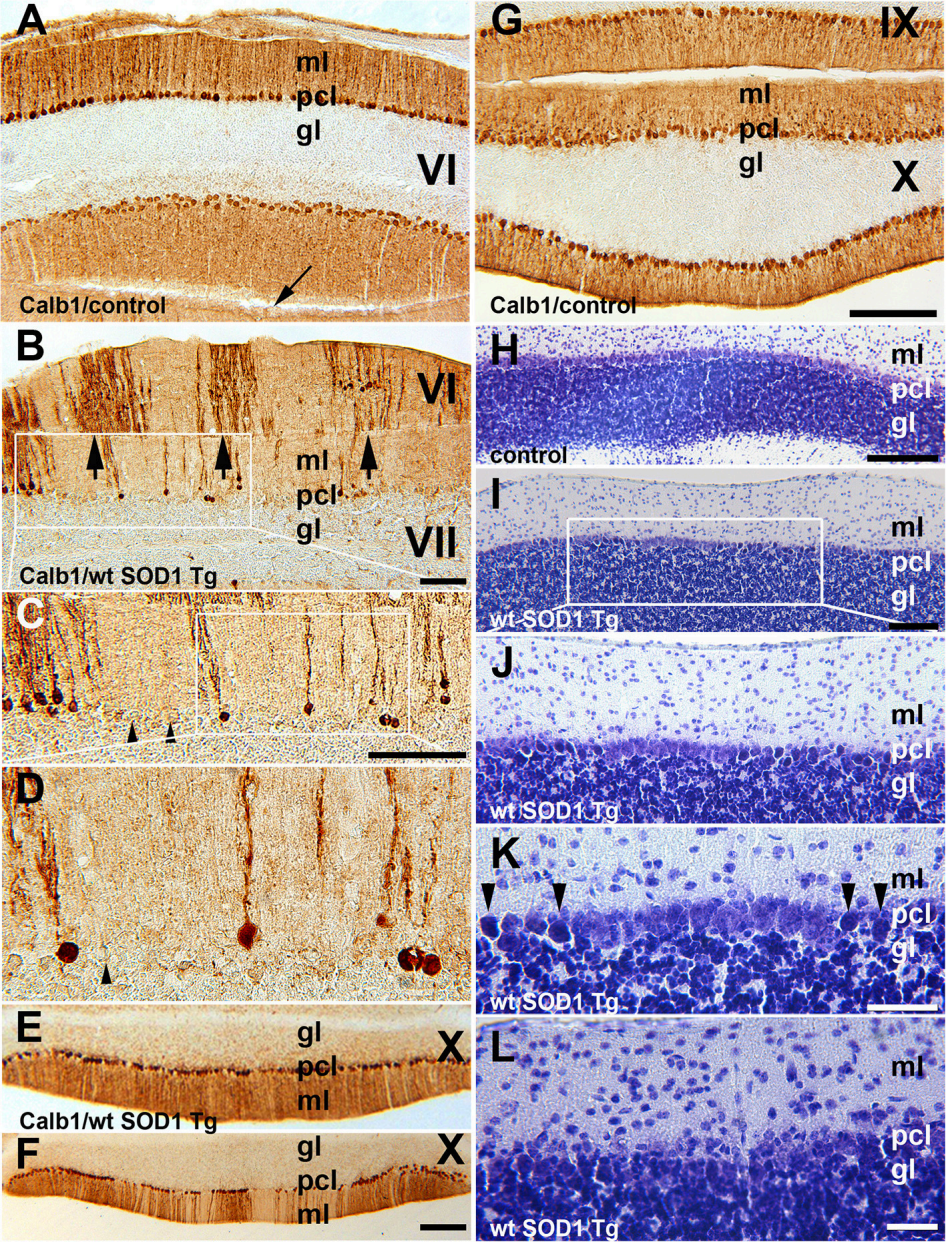
**Calb1 expression is down-regulated in Purkinje cells in the CZ and NZ of wt ***SOD1*** Tg mice. (A,G)** A transverse section through the lobules VI (CZ) and IX/X (NZ) immunostained with Calb1 specify uniform expression in Pcs in entire cerebellar cortex of 8-month-old control C57BL/6 mouse cerebellum. Arrow indicates “primary fissure” in between lobules V and VI. **(B–D)** A transverse section through lobules VI/VII (CZ) immunostained with Calb1 in the cerebellum of 5-month-old wt SOD1 Tg mice. A subset of Purkinje cell soma and dendrites lacking Calb1 expression, and alternating with Calb1^+^ Purkinje cells (arrows) that are symmetric about the midline are shown. **(B)** Is a higher magnification of the box in **(A)**. **(C)** Is higher magnification of the box in **(B)**. **(E)** A transverse section through lobule X (NZ) immunostained with anti Calb1 in a 5-month-old wt *SOD1* Tg mice cerebellum. **(F)** A transverse section through lobule X (NZ) immunostained with anti Calb1 in an 8-month-old wt *SOD1* Tg mice cerebellum. **(H)**. A transverse section through lobule X (NZ) prepared using cresyl violet staining (Nissl staining) in a 5-month-old control mouse cerebellum. Large Purkinje cell bodies with light staining forms a monolayer between the molecular layer (ml) and granular layer (gl). **(I–K)** A transverse section through lobule X (NZ) prepared using cresyl violet staining in an 8-month-old wt *SOD1* Tg mice cerebellum. Nissl-stained dark Purkinje cells body scattered in Purkinje cell layer between ml and gl **(I)**. Nissl-stained dark Purkinje cell bodies were smaller and condensed **(J)**, and they are indicated by the arrowhead in the higher magnification in “**(K)**.” **(L)** A transverse section through lobule VII prepared using cresyl violet staining in an 8-month-old wt *SOD1* Tg mice cerebellum. The putative Purkinje cell layer lacks Purkinje cell bodies and may indicate cell death. Gl, granular layer; pcl, Purkinje cell layer; ml, molecular layer. Scale bar = 250 μm in **(B)**; 500 μm in **(C)**; 250 μm in **(F)** (applies to **E,F**); 250 μm in **(G)** (applies to **A,G**); 250 μm in **(H,I)**; 100 μm in **(K,L)**.

In the normal mouse cerebellum, the uniform distribution of Calb1 immunopositive cell bodies in the Purkinje cell layer and processes in the molecular layers are shown in lobule VI and IX–X (Figures 2A,G). However, in the wt *SOD1* Tg mice cerebellum, immunostaining with Calb1 revealed symmetric gaps between Purkinje cells about the midline (Figures 2B–F). To identify whether Purkinje cell death is responsible for the Calb1 expression pattern in the wt *SOD1* Tg mice cerebellum, we performed cresyl violet staining (Nissl staining, which is staining in the rough endoplasmic reticulum and free ribosomes in neurons; Figures 2H–L). Nissl staining of cerebellar sections from the CZ and NZ show differences between normal Purkinje cells (light staining in neurons; Figure 2H) and the degenerating cells with Nissl-stained dark soma in the wt *SOD1* Tg mice cerebella (Figures 2I–K). The Calb1 immunonegative Purkinje cells or degenerating Purkinje cells (about 58% of total Purkinje cells in CZ) were condensed with Nissl staining and therefore they appeared darker compared to normal healthy cells (Figures 2I–K). Nissl staining showed a lack of obvious Purkinje cell soma; this could suggest cell death or severe neuron atrophy (Figure 2L; Tajiri et al., 2004; Ooigawa et al., 2006). Therefore, Calb1 down-regulation and probably degenerating Purkinje cells are responsible for the absence of immunoreactivity that forms the array of parasagittal gaps in the wt *SOD1* Tg mice cerebellar cortex. Calb1 down-regulation was not present in all cerebellar zones, but instead, it followed a spatio-temporal pattern. At 5 months of age, it was prominent in the CZ (Figure 2B) and at 8 months in the NZ (Figure 2F), but it was not observed in the AZ/PZ (data not shown).

**Figure 3:**
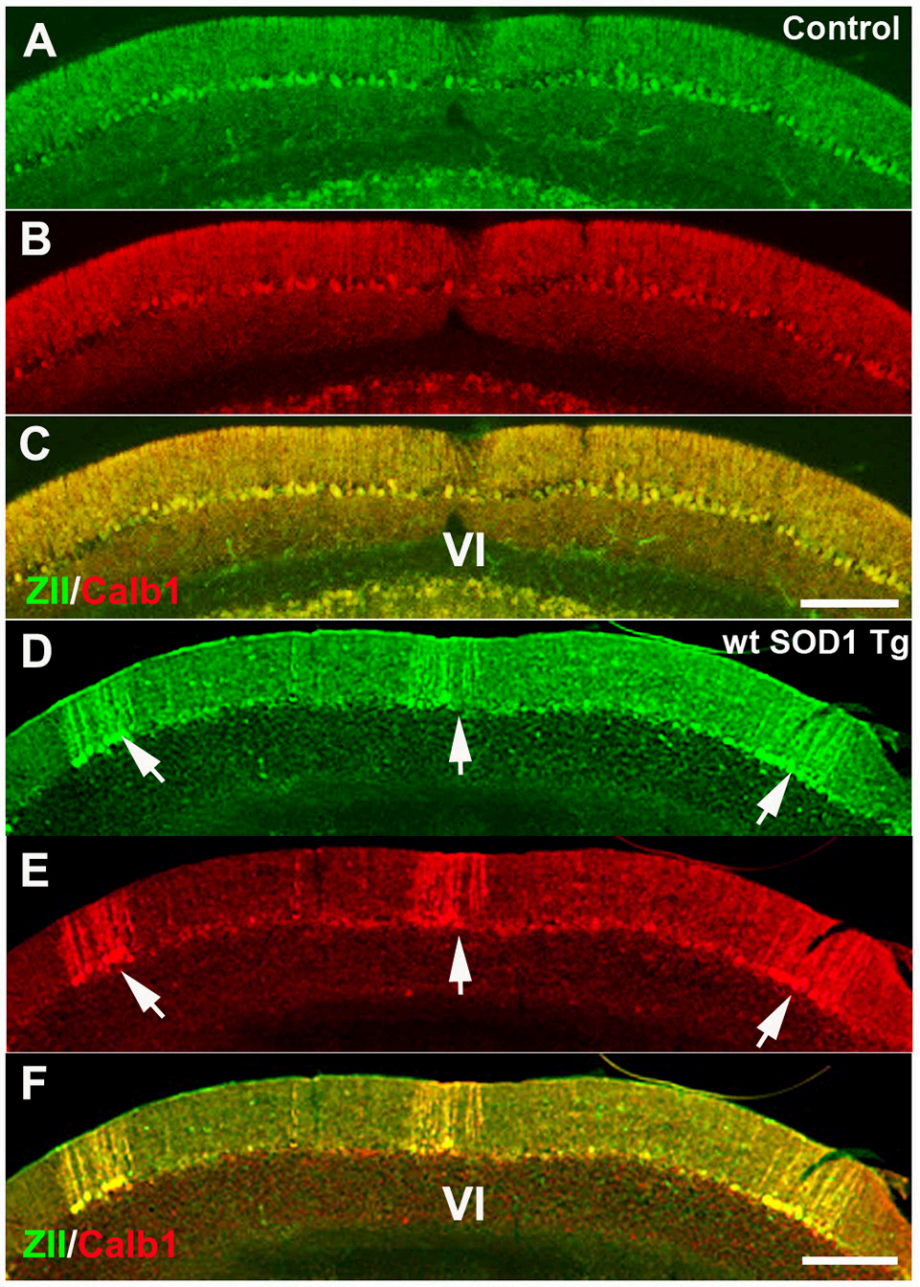
**ZII expression is down-regulated in Purkinje cells in the CZ. (A–C)** ZII expression is uniform in the CZ (green), and Calb1 is expressed (red) in all Purkinje cells in the control mouse cerebellum. **(D–F)** ZII expression is down-regulated in the CZ, similar to Calb1 expression, and gives the appearance of patterned parasagittal stripes in the CZ in an 8-month-old wt *SOD1* Tg mice cerebellum. Scale bar = 250 μm in **(C)** (applies to **A–C**); 250 μm in **(F)** (applies to **D–F**).

We used ZII antibody to determine whether expression of the zone- and stripe marker was affected in wt *SOD1* Tg mice. Immunostaining with ZII, which is expressed uniformly in the CZ of the normal mouse cerebellum (Figures 3A–C), revealed a symmetrical array of parasagittal stripes (Figure 3D) in the wt *SOD1* Tg mice cerebellum, which is co-labeled with the Calb1 expression stripes in lobule VI/VII of the surviving Purkinje cells (Figures 3E,F).

**Figure 4:**
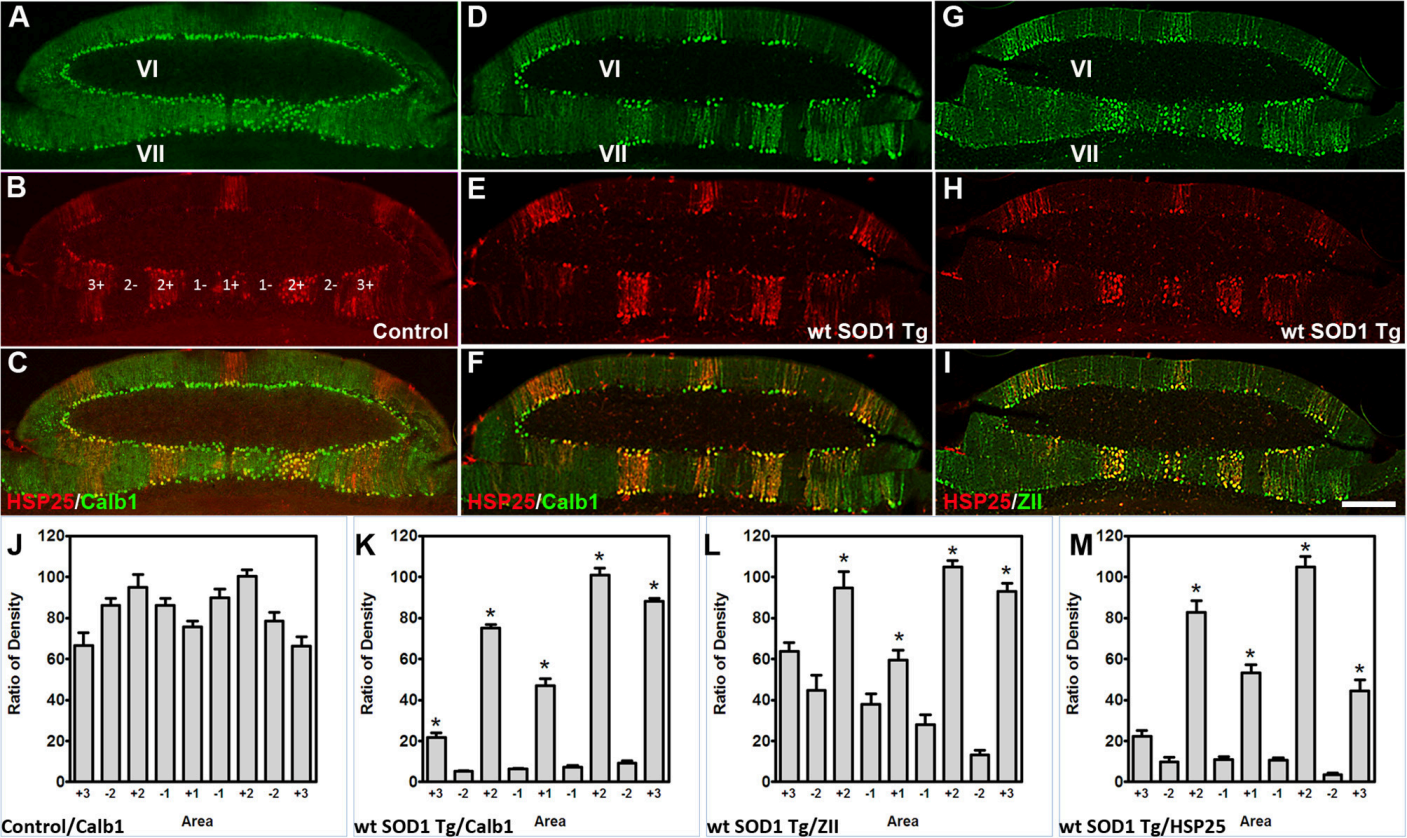
**Calb1 and ZII expression is compared with HSP25 in the CZ of wt ***SOD1*** Tg mice. (A–C)** Double immunostaining of HSP25 and Calb1 was performed on transverse sections through the CZ in a normal mouse. Calb1 was uniformly expressed in **(A)**, while lobules VI/VII showed five parasagittal bands in the vermis that were symmetric about the midline **(B)**. There was one midline and two on each side that were merged in **(C)**. **(D–F)** Double labeling of Purkinje cells in the transverse section of the cerebellar CZ. Calb1 (green) and HSP25 (red) immunofluorescence in the wt *SOD1* Tg mice. Calb1 is expressed in parasagittal stripes in the lobule VI/VII of wt *SOD1* Tg mice **(D)**. HSP25 is expressed in five parasagittal stripes in lobule VI/VII in *SOD1* Tg mice **(E)**. Double staining shows a strong correlation between HSP25 and Calb1 expression **(F)**. **(G–I)** Double labeling of Purkinje cells in the transverse section of the cerebellar CZ. ZII (green) and HSP25 (red) immunofluorescence in the wt *SOD1* Tg mice. ZII is expressed in parasagittal stripes in the lobule VI/VII of wt *SOD1* Tg mice **(G)**. HSP25 is expressed in five parasagittal stripes in lobule VI/VII in wt *SOD1* Tg mice (H). Double staining shows a strong correlation between HSP25 and ZII expression **(I)**. **(J–M)** The integrated density was measured within multiple selected area of images that immunostained with Calb1 (**J**; control, **K**; wt *SOD1* Tg), ZII (**L**; wt *SOD1* Tg), and HSP25 (**M**; wt *SOD1* Tg). The HSP25 immuno-positive/negative Purkinje cells are shown with “+” and “−” numbers symmetric about the midline. Scale bar = 250 μm in **I** (applies to **A–I**). Asterisk (^*^) show a significant difference between groups (*P* < 0.005).

**Figure 5:**
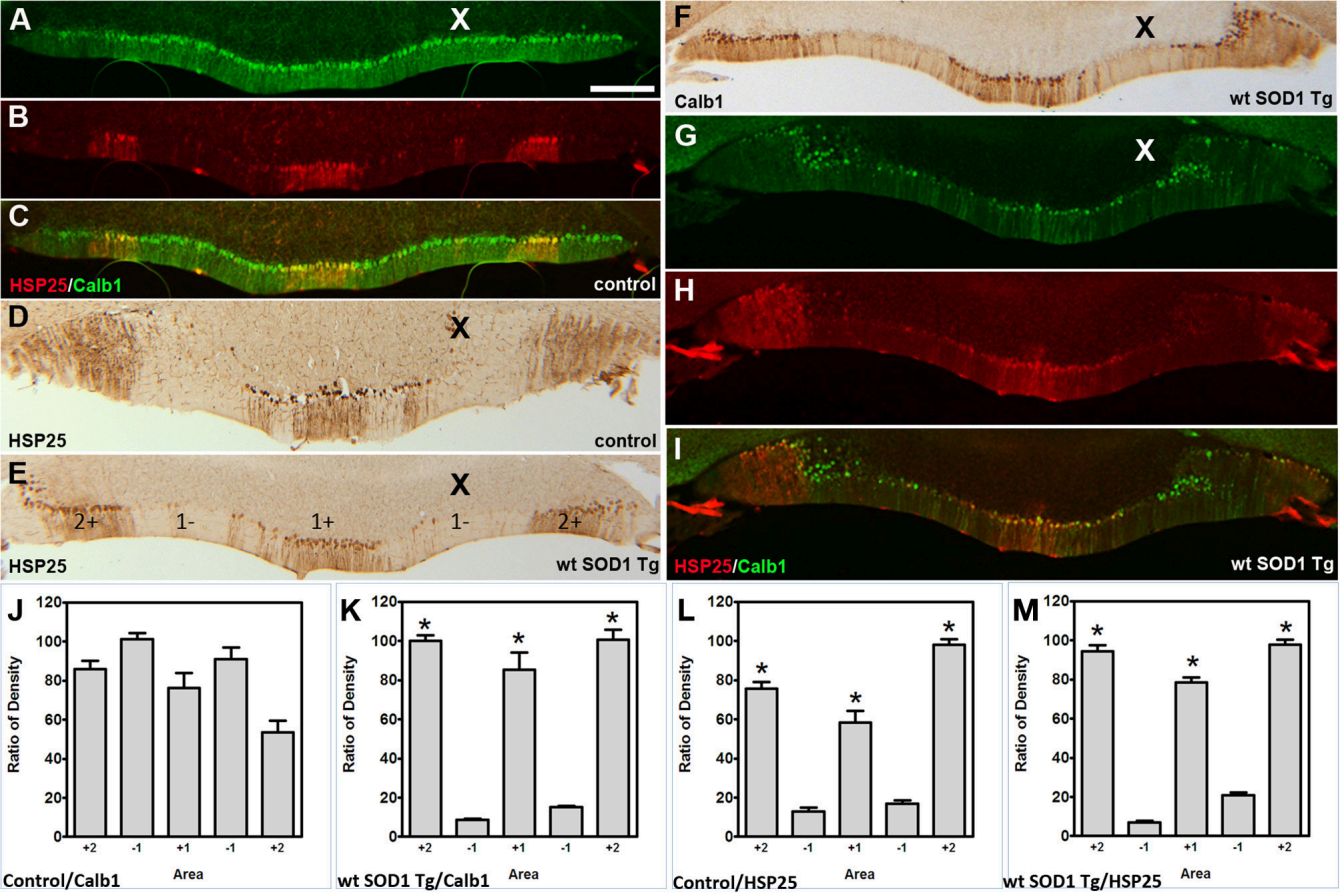
**Calb1 and ZII expression compared with HSP25 in the NZ of wt ***SOD1*** Tg mice. (A–C)** Double staining for Calb1 (green) and HSP25 (red) in the NZ of the control mouse cerebellum. **(D,E)** Peroxidase staining of HSP25 shown in the NZ of the cerebellum of control mice **(D)** and wt *SOD1* Tg mice **(E)**. **(F)** Peroxidase staining of Calb1 shown in the NZ of the wt *SOD1* Tg mice cerebellum. **(G–I)** Double staining for Calb1 (green) and HSP25 (red) in the NZ of the wt *SOD1* Tg mice cerebellum reveals that Calb1 expression mirrors the pattern of HSP25. **(J–M)** The integrated density was measured within multiple selected areas of images that immunostained with Calb1 (**J**; control, **K**; wt *SOD1* Tg) and HSP25 (**L**: control, **M**; wt *SOD1* Tg). The HSP25 immuno-positive/negative Purkinje cells are shown with “+” and “−” numbers symmetric about the midline. Scale bar = 250 μm in **(A)** (applies to **A–I**). Asterisk (^*^) show a significant difference between groups (*P* < 0.005).

The Calb1 and ZII immunopositive stripes in the wt *SOD1* Tg mice are reminiscent of the HSP25 expression pattern in the CZ of the control cerebellum (Figures 4A–C). To determine whether the Calb1 expression pattern is similar to that of HSP25 expression in the CZ of the wt *SOD1* Tg cerebellum, we performed double immunostaining for Calb1 and HSP25. The Calb1 immunostaing showed that the pattern of surviving Purkinje cells co-labeled and aligned with the HSP25 expression pattern in the CZ (Figures 4D–F). To confirm this expression pattern, further immunohistochemistry was performed on the transverse cerebellar sections from wt *SOD1* Tg mice to investigate the ZII expression in the CZ, where Calb1 was down-regulated. Double staining for ZII and HSP25 revealed that ZII is strongly expressed in alignment with the HSP25 immunopositive bands and it was not present in HSP25 immunonegative bands in the vermis of lobules VI and VII (Figures 4G–I). To quantify the intensity of expression pattern, we measured Calb1, ZII, HSP25 expression in the vermis of lobule VII (*N* = 3). The average pixel intensity is shown by bar graph representing level of Calb1 expression in control group (Figure 4J). Each selected area is corresponding to the pattern of HSP25 expression in which (“+”) and (“−”) numbers indicate HSP25^+/−^ symmetric about the midline (1+; Figure 4J). While high and low intensity of Calb1 and ZII expression in wt *SOD1* Tg in the vermis of lobule VII (Figures 4K,L) indicate the stripe pattern comparable with HSP25 expression (Figure 4M). Similar to the CZ, HSP25 was expressed in the NZ in parasagittal stripes. This has been shown by double labeling of Calb1 and HSP25 in the NZ (Figures 5A–D). HSP25 expression in the NZ of the wt *SOD1* Tg mice was similar to the control cerebellum (Figures 5D–E). Calb1 was expressed in parasagittal stripes, aligned with the HSP25 immunopositive Purkinje cells in lobule X (Figures 5F–I). The intensity of expression has been measured in the vermis of lobule X for Calb1 in comparison with HSP25 expression (*N* = 3). There were five stripe pattern of HSP25 expression in lobule X in mouse cerebellum in which immunopositive (“+”) and immunonegative (“−”) numbers represent symmetry about the midline (1+). The average pixel intensity is demonstrated by bar graph representing level of Calb1 expression in control group (Figure 5J). The alternate high and low intensity of Calb1 expression in wt *SOD1* Tg in lobule X (Figure 5K) indicate the stripe pattern which is comparable with HSP25 expression in both control and wt *SOD1* Tg (Figures 5L,M).

### Calb1 was down-regulated in a patterned manner in wt *SOD1* Tg mice with unsteady gait

**Figure 6:**
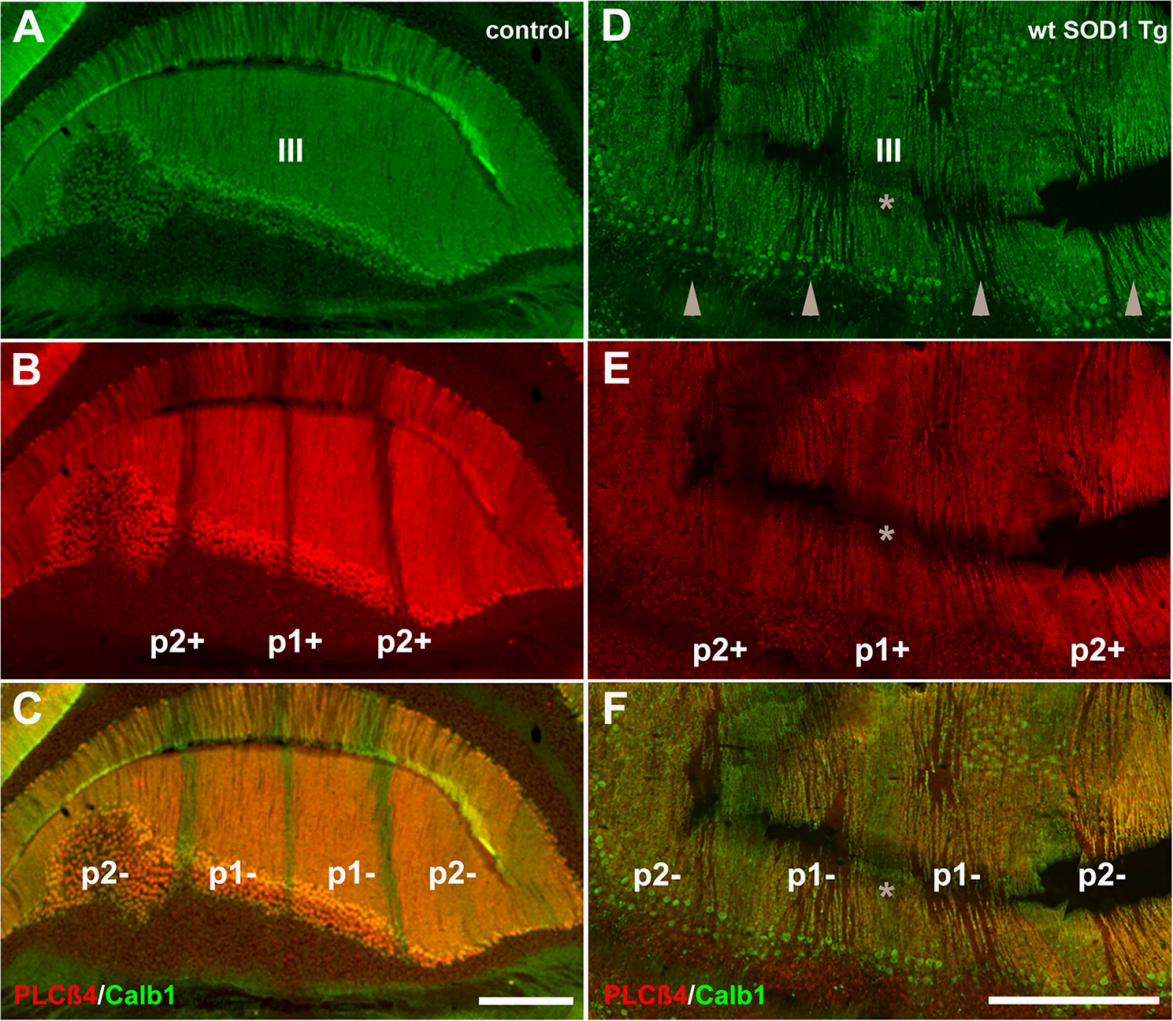
**Comparison of Calb1 and PLCβ4 expression in the control (A–C)** and ataxic wt *SOD1* Tg mice **(D**–**F)** in the AZ cerebellar transverse section. **(A–C)** Calb1 (green) and PLCβ4 (red) double staining shows uniform expression of Calb1 **(A)** and a parasagittal stripe pattern for PLCβ4 expression in lobule III of the normal mouse cerebellum. **(D–F)** Calb1 expression in the wt *SOD1* Tg mice (green) mouse cerebellum shows down-regulation in a subset of Purkinje cells that are symmetrical about the midline (indicated by asterisk). PLCβ4 immunostaining shows down-regulation in a subset of Purkinje cells that are located in middle of the P1-stripe (ZII^−^/PLCβ4^+^) and medial to the P2-stripe. Scale bar = 250 μm in **(C)**; (applies to **A–C**); 250 μm in **(F)** (applies to **D–F**).

**Figure 7:**
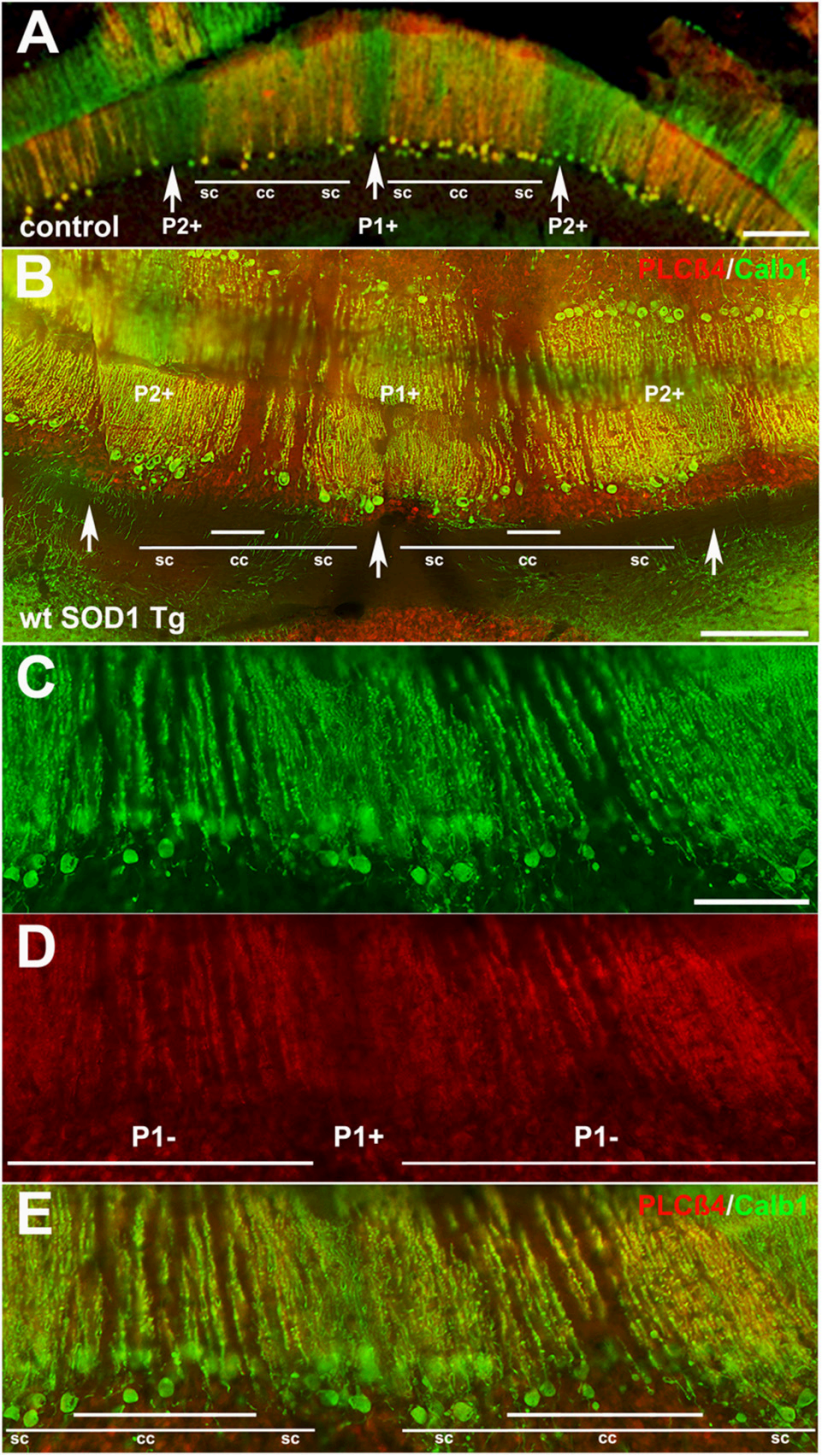
**Detailed information for Calb1 and PLCβ4 expression, presented at a higher magnification, in the control (A)** and ataxic wt *SOD1* Tg mice **(B–E)** in the AZ cerebellar transverse section. **(A)** PLCβ4 stripes between P1^+^ and P2^+^ (arrow) receive somatosensory afferent input that is arranged from medial to lateral, as follows: spinocerebellar (sc), cuneocerebellar (cc) and spinocerebellar afferents (e.g., Marzban et al., 2007). **(B–E)** Calb1 and PLCβ4 expression in the ataxic wt *SOD1* Tg mice **(B–E)** in the AZ cerebellar transverse section shows that the middle part of the PLCβ4^+^ afferents are either down-regulated (both Calb1/PLCβ4) or possibly a degenerated subset of Purkinje cells that receive cunesocerebellum afferent input. Scale bar = 100 μm in **(A,B)**; 50 μm in **(C)** (applies to **C–E**).

Behavioral observations showed aggressiveness in all transgenic and 15 mice required individual housing. Two out of the 15 individually-housed mice developed severe movement disorder and unsteady gait such as ataxia-like behaviors in older adults. No abnormalities were observed in their littermate of both wt *SOD1* Tg and control mice. The pattern of Calb1 down-regulation in the CZ and NZ of the wt SOD1 Tg mice with unsteady gait resembles the wt *SOD1* Tg mice cerebellum. However, an additional remarkable pattern change was seen in the AZ of the wt *SOD1* Tg mice with unsteady gait. PLCβ4 immunostaining was used to determine the pattern of Calb1 down-regulation in the wt *SOD1* Tg mice with unsteady gait cerebellum. In comparison to control and wt *SOD1* Tg with normal gait mice cerebellum (Figures 6A–C, 7A), wt *SOD1* Tg mice with unsteady gait cerebellum has a missing subset of PLCβ4^+^/ZII^−^ Purkinje cell stripes (Figures 6D–F, 7B–E). It seems that down-regulation occurs in the middle of the PLCβ4^+^ stripe (P1−), between the p1+ and P2+ bands and also lateral to the P2^+^ band [medial subset of PLCβ4^+^ Purkinje cells (P2−)] in the AZ (Figures 6D, 7B). A higher magnification of the PLCβ4^+^ stripe (P1−) between the p1+ and P2+ bands, revealed a low density of Purkinje cells rather than down-regulation of both Calb1 and PLCβ4 expression (Figures 7C–E). However, this was not observed in the PZ (data not shown).

## Discussion

The wt *SOD1* Tg mice cerebellum was examined to determine whether the compartmentation of the Purkinje cells had been altered. The Calb1 and ZII expression was changed prominently in the CZ and NZ of the wt *SOD1* Tg mice cerebella with or without unsteady gait. Calb1 and ZII expression was down-regulated and Purkinje cells with Calb1^+^/ZII^+^ were left in a stripe pattern that closely resembles and aligns with the parasagittal stripe pattern in the CZ and NZ, which is revealed by HSP25. The wt *SOD1* Tg mice with unsteady gait cerebellum showed an additional phenotype that had Calb1 down-regulation in a subset of Purkinje cells in the PLCβ4^+^/ZII^−^ stripes in the AZ, which may explain the cause of gait unsteadiness. The results of this study on wt *SOD1* Tg mice, 5–8 months old, show TWO interesting issues that will be discussed: (1) down-regulation of Calb1/ZII expression beginning and prominent in the CZ and NZ; and (2) the pattern of Calb1/PLCβ4 down-regulation in the AZ in wt *SOD1* Tg mice with unsteady gait.

The cytoarchitecture in the cerebellar cortex forms an array of transverse zones and parasagittal stripes. The symmetric parasagittal stripes that are formed by heterogeneous Purkinje cells express various types of genes and proteins. The most well-known of these genes and proteins are ZII, phospholipase Cß4 and the small HSP25 (Hawkes, 1997; Oberdick et al., 1998; Armstrong and Hawkes, 2000; Sarna et al., 2006).

Purkinje cell degeneration is a complex process in neurodevelopmental disorders of the cerebellum (Sarna and Hawkes, 2003; Sarna et al., 2003; Marzban et al., 2014). In the cerebellum, several studies involving mutant mice have shown that there is a general spatio-temporal pattern of Purkinje cell degeneration. Wassef et al. investigated Purkinje cell degeneration in the nervous (nr), Purkinje cell degeneration (pcd), and tambaleante (*tbl*) mutant mice. They have shown severe postnatal Purkinje cells death in nr, Pcd, and tb1 cerebellum with the pattern of surviving Purkinje cells were symmetric about the midline (Wassef et al., 1987). In addition, in the ataxic sticky (*sti/sti*) and *NPC* mutant mice, the Purkinje cell loss were not random, but aligned with zone and stripes pattern and symmetric with midline (Sarna and Hawkes, 2003, 2011). Recently, Cerminara et al. have summarized several patterned cerebellar neurodegeneration disorders which are associated with gene mutations (Cerminara et al., 2015). In most cases, specific Purkinje cell populations, which are located within the AZ and PZ, degenerate earlier than those from the CZ and NZ. In addition, PLCβ4^+^/ZII^−^ are more susceptible to Purkinje cell death and PLCβ4^−^/ZII^+^ are resistant and survive longer (Sarna and Hawkes, 2003, 2011; Sarna et al., 2003; Vogel et al., 2007; Duffin et al., 2010). However, in wt *SOD1* Tg mice, Calb1 down-regulation target a specific subset of Purkinje cells in the CZ and NZ. The non-affected Purkinje cells that are immunoreactive with Calb1 in the CZ and NZ of wt *SOD1* Tg mice appeared parasagittally striped, and are aligned with the HSP25 stripes in the CZ and NZ.

The decreased number of Calb1-positive neurons in the cerebellum of wt *SOD1* Tg mice may indicate that Purkinje cells are degenerating or degenerated (e.g., Ooigawa et al., 2006). An explanation for the Purkinje cell degeneration is a disturbance in calcium homeostasis (Lally et al., 1997; Phillips et al., 1999). Calb1 may act as a buffer for neuronal calcium (Ng and Iacopino, 1995) and changing the calcium level in neurons may result in activation of cascades relevant to cell death and consequently, neurodegeneration. In Alzheimer's patients, it has been shown that the number of Calb1-positive neurons decreases, and these cells have been shown to shrink (Ichimiya et al., 1988; Hof and Morrison, 1991; Kurobe et al., 1992; Lally et al., 1997). In the cerebellum of seizure-sensitive gerbils, there is a decrease in the Calb1 immunoreactivity and a loss of Purkinje cells (Kang et al., 2002). Purkinje cell degeneration may result from an increase in intracellular Ca^2+^ levels, which may trigger molecular events related to neuronal degeneration, conceivably by stimulating calcium-dependent enzymes (e.g., Choi and Rothman, 1990). Therefore, Calb1 is a crucial protein member of the calcium binding proteins, which regulate intracellular calcium levels in neurons and play an important role in the nervous system (Baimbridge et al., 1982). Further, Calb1 is a sensitive immunohistochemical marker of cerebellar neurotoxicity (Haworth et al., 2006). Since Calb1 plays an important role in neurons, down-regulation of this protein is associated with cellular injury. Although down-regulated Calb1 may present randomly and asymmetrically in some cases, the pattern and symmetry of Calb1 downregulation about the midline may suggest Purkinje cell degeneration with a fundamental pathological process during cerebellar neurodevelopmental disorders.

In the transgenic mouse with human SOD1 expression, Calb1 expression was down-regulated, suggesting that the human *SOD1* gene and/or its product interfere with normal Purkinje cell function, such as Calb1 expression. It has been reported that human SOD1 overexpression can cause mitochondrial vacuolization, axonal degeneration and premature motor neuron death (Jaarsma et al., 2000). It is possible that SOD1 is overexpressed in wt *SOD1* Tg mice Purkinje cells, and that the protein plays a toxic role and interferes with normal cell functions, leading to a reduction in Calb1 expression. It has been shown that Calb1 is reduced by 39–55% in the cerebellum when a toxin such as amphetamine was administered to rats (Yin et al., 2010). In another study, chronic administration of morphine to rats decreased the Calb1 immunoreactivity in a subset of Purkinje cells (Garcia et al., 1996). This could be the case with the SOD1 protein overexpression and as a result of SOD1 protein toxicity, the subset of Purkinje cells lack Calb1 expression and may degenerate. Furthermore, previous investigations showed that Calb1-expressing neurons have a higher survivability under ischemia and excitotoxicity conditions (Mattson et al., 1991). Also, Calb1 overexpression has been shown to have protective role striatal neurons in transient focal cerebral ischemia (Yenari et al., 2001). In addition, astrocytes also express Calb1 when brain injury occurs (Mattson et al., 1995). Therefore, it is not surprising to observe that Calb1-expressing cells have better survivability than those that lack Calb1 expression; Purkinje cells that lack Calb1 are susceptible to degeneration. It is well-evident that glutamatergic system plays a key role in excitotoxicity under pathological conditions. Excitatory amino acid transporter (EAAT) 4, which is found in Purkinje cells (Inage et al., 1998; Welsh et al., 2002), is mostly expressed in ZII^+^ cells and causes more resistance to excitotoxic damage in these cells (Welsh et al., 2002). EAAT4 helps to reduce extracellular glutamate concentrations, and therefore EAAT4/ZII-positive cells have greater survivability. However, our data with *SOD1* Tg mice showed down-regulation (preceding degeneration) even with EAAT4-positive Purkinje cells. Thus, excitotoxicity may not be an underlying reason for the observed patterned Purkinje degeneration.

An interesting observation was that Purkinje cells expressed Calb1 and ZII in parasagittal stripes in the CZ and NZ, which mirrored HSP25 expression. This suggests differences between HSP25-immunopositive and -immunonegative Purkinje cells. HSP25 function is unclear in the cerebellum, but in non-neuronal cell lines, it is a molecular chaperone (Jakob et al., 1993) that regulates actin filament organization and stabilization during oxidative stress (Lavoie et al., 1993), regulates anti-oxidative activity (Mehlen et al., 1996a,b), protects cells and improves cell survival (Sarna and Hawkes, 2003). HSP25's association with protecting against cell degeneration, preferentially with the surviving cells, is unclear in the cerebellum; however, it functions as a molecular chaperone and regulates anti-oxidative activities in cells. It would not be surprising if HSP25 had the same role in a subset of Purkinje cells and regulating anti-oxidation activities, thereby returning the Purkinje cells to healthy cells. This is one explanation for the normality of Purkinje cells in the HSP25^+^ regions. Previous studies have observed that wt human SOD1 is toxic to neurons when it is overexpressed. Further studies are needed to determine whether this adverse property of wt SOD1 derives from posttranslational modifications, as was previously suggested (Ezzi et al., 2007; Chen et al., 2012). Our data raises a concern about previous studies on the toxicities of ALS-linked *SOD1* mutations because in those studies, the wt *SOD1* Tg mice used as controls (Wong et al., 1995; Kong and Xu, 1998). Wt SOD1 is toxic to Purkinje cells; it down-regulates Calb1 and could potentially be toxic to motor neurons as well. Conclusions in previous studies that used the wt *SOD1* animal as a control may therefore need to be revisited.

It seems that the progression of Purkinje cell phenotypic alterations and probably degeneration is faster in a subpopulation of the wt *SOD1* Tg mice and this leads to appearance of movement disorders and unsteady gait behavior in mice. We did not perform a detailed motor analysis—however, gait unsteadiness was apparent in two wt *SOD1* Tg mice at 8 months of age, and were accompanied by Calb1 down-regulation in the subset of PLCß4^+^ Purkinje cells in the AZ. The reason for unsteady gait may be because of the Calb1 down-regulation in specific subsets of PLCß4^+^ Purkinje cells that receive cuneocerebellar projections, which are a somatosensory pathway (Marzban et al., 2007). It has been suggested that selective deletion of the Calb1 gene can lead to behavioral and cellular differences in Purkinje cells and, ultimately, permanent deficits in motor-coordination and proprioceptive sensory processing. Development of ALS is a result of over-expression and aggregation of SOD1 proteins (Rosen et al., 1993; Blokhuis et al., 2013). In our study with wt *SOD1* Tg mice, the protein may have aggregated in the Purkinje cells and interfered with cell function, leading to down-regulation of Calb1 and other proteins, which was ultimately followed by Purkinje cell degeneration. However, further research is needed at the molecular level, which can be measured using western blotting, and compared with control mice to investigate possible over-expression of SOD1 and Purkinje cell degeneration.

## Conclusions

We found that wt *SOD1* Tg mice showed abnormalities in the cerebellum compared to normal mice. The Calb1 down-regulation is patterned according to the fundamental architecture of the cerebellum. However, wt *SOD1* Tg mice showed a unique pattern in which Calb1 down-regulation began in the CZ and progressed to the NZ, which is common in both with and without unsteady gait groups. Although all Purkinje cells in both the CZ and NZ are ZII immunopositive, it is tempting to speculate that a subset of Purkinje cells in the AZ ZII^+^/HSP25^−^ stripes is affected. However, it seems that Calb1 down-regulation in the AZ occurs in a subset of Purkinje cells in the ZII^−^/PLCβ4^+^ stripes. The ZII^−^/PLCβ4^+^ Purkinje cells are targeted by somatosensory afferents, particularly cuneocerebellar afferents, and may explain unsteady gait. This suggests that, although the Purkinje cell pathologic pattern in wt *SOD1* Tg mice reflects the fundamental cytoarchitecture of the cerebellum, the pattern in each zone manifests differently.

## Author contributions

Designed the experiments: HM. Performed the experiments: PA, NA, XJ, MR, and XZ. Analyzed the data and wrote the paper: PA, NA, XJ, MB, XZ, BY, MD, JK, and HM.

### Conflict of interest statement

The authors declare that the research was conducted in the absence of any commercial or financial relationships that could be construed as a potential conflict of interest.

## Supplementary material

Supplementary Figure 1 **SOD1 expression is localized in Purkinje cell layer of wt ***SOD1*** Tg mice and human cerebella. (A)** A transverse cryostat sections through the lobule VI of the 8 month control mouse cerebellum immunostained with anti-SOD1 shows lack of SOD1 expression in Purkinje cell soma; shown at a higher magnification in “**(a)**.” **(B,C)** A transverse section through the cerebellum of the 8-month-old wt *SOD1* Tg mice immunostained using anti-SOD1 shows weak expression in the molecular layer (B) and intense expression in Purkinje cell soma (**b,C**, arrows). **(D,d,E)** A transverse paraffin sections through the human cerebellar cortex immunostained with anti-SOD1 displays presence of SOD in Purkinje cell soma (arrow) and dendrite (arrow head). **(F,f)** A transverse paraffin sections through the postmortem ALS patient cerebellar cortex immunostained using anti-SOD1 shows expression in Purkinje cell soma (arrow) and dendrite (arrow head) and also probably in GABAergic interneurons of the molecular layer. A SOD1 immunostained Purkinje cell is shown at a higher magnification in “**(f)**.” Scale bar = 100 μm in **(F)** (applies to **A,B,D,F**); 50 μm in **(F)** (applies to **C–E**).

## Abbreviations

ALS: Amyotrophic lateral sclerosis
Calb1: Calbindin 1
HSP: Heat shock protein
NZ: Nodular zone
PBS: Phosphate buffer saline
Cβ4: PLCβ4: Phospholipase
PZ: Posterior zone
SOD1: Superoxide dismutase 1
Tg mice: Transgenic mice
WT: Wild type
ZII: Zebrin II.
