# Supplementary figures and images for: Overexpression of Human SOD1 Leads to Discrete Defects in the Cerebellar Architecture in the Mouse
Source: Front Neuroanat. 2017 Mar 29;11:22. doi: 10.3389/fnana.2017.00022 (PMC5372795; doi:10.3389/fnana.2017.00022)

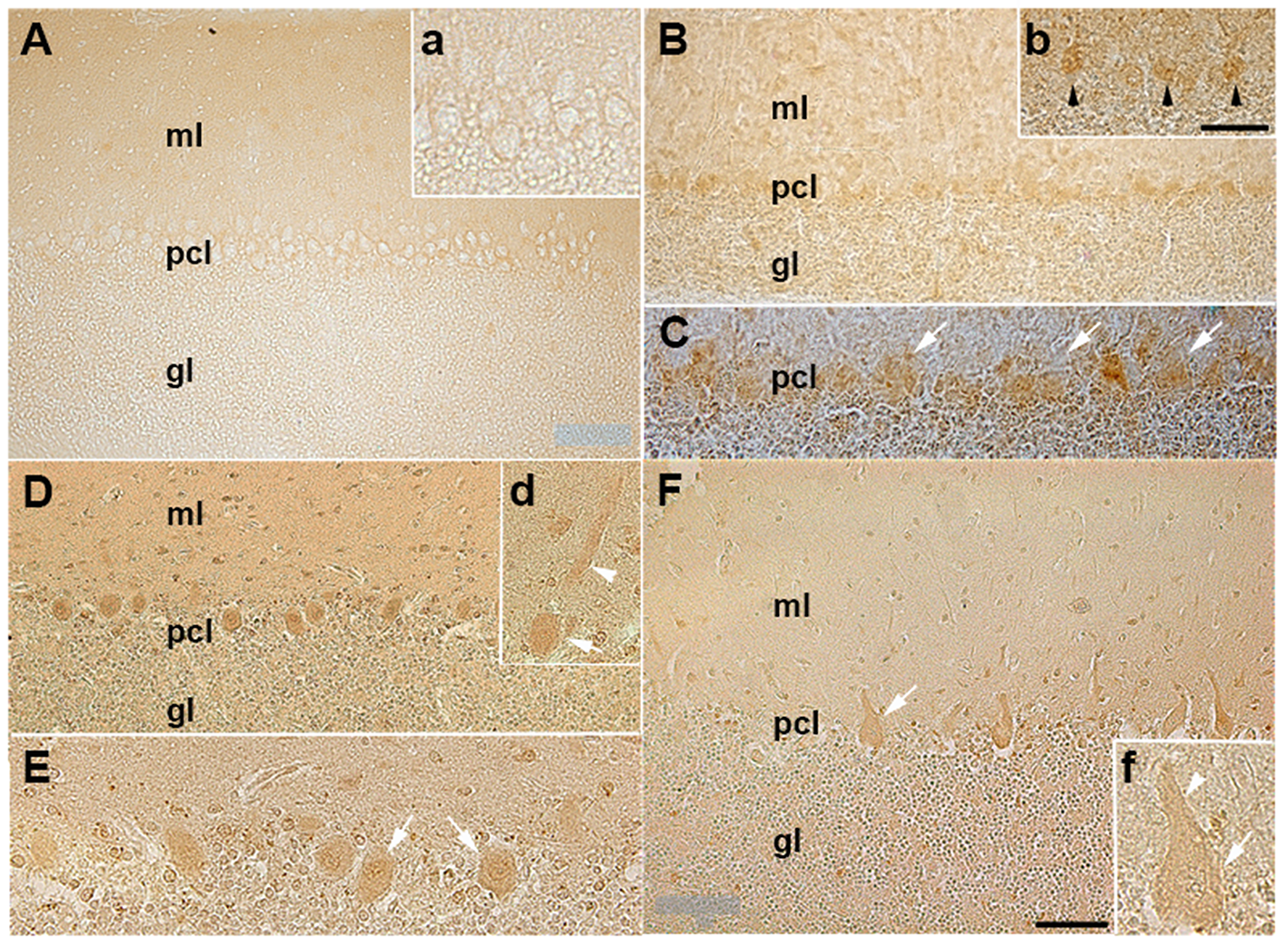

Supplement: Supplementary Figure 1 — SOD1 expression is localized in Purkinje cell layer of wt SOD1 Tg mice and human cerebella. (A) A transverse cryostat sections through the lobule VI of the 8 month control mouse cerebellum immunostained with anti-SOD1 shows lack of SOD1 expression in Purkinje cell soma; shown at a higher magnification in “(a).” (B,C) A transverse section through the cerebellum of the 8-month-old wt SOD1 Tg mice immunostained using anti-SOD1 shows weak expression in the molecular layer (B) and intense expression in Purkinje cell soma (b,C, arrows). (D,d,E) A transverse paraffin sections through the human cerebellar cortex immunostained with anti-SOD1 displays presence of SOD in Purkinje cell soma (arrow) and dendrite (arrow head). (F,f) A transverse paraffin sections through the postmortem ALS patient cerebellar cortex immunostained using anti-SOD1 shows expression in Purkinje cell soma (arrow) and dendrite (arrow head) and also probably in GABAergic interneurons of the molecular layer. A SOD1 immunostained Purkinje cell is shown at a higher magnification in “(f).” Scale bar = 100 μm in (F) (applies to A,B,D,F); 50 μm in (F) (applies to C–E). [file Image1.tif]
